# Supplementary material for: People’s perceptions of, willingness-to-take preventive remedies and their willingness-to-vaccinate during times of heightened health threats
Source: PLoS One. 2022 Feb 2;17(2):e0263351. doi: 10.1371/journal.pone.0263351 (PMC8809555; doi:10.1371/journal.pone.0263351)
Supplement: S1 Table — N, number of participants; M, mean; SD, standard deviation; 1 due to low case number ‘other’ were excluded for the statistical test. (DOCX) [file pone.0263351.s001.docx]

**S1 Table. Sociodemographics separated by condition and statistical test of similarity in sociodemographics among the four conditions.**

|  | **high salience** |  | **low salience** |  | **Statistical test** |
| --- | --- | --- | --- | --- | --- |
|  | **natural remedy** | **synthetic remedy** | **natural remedy** | **synthetic remedy** |  |
| **Total *N*** | 126 | 122 | 115 | 123 |  |
| **Age (*M*, *SD*)** | 44.6 (15.7) | 44.6 (14.2) | 46.2 (14.8) | 45.9 (14.8) | *F*(3, 482) = 0.38, *p* = .767 |
| **Gender *N***^1^ |  |  |  |  |  |
| male | 59 | 67 | 51 | 63 | Χ^2^(3) = 2.98, *p* = .395 |
| female | 65 | 55 | 64 | 60 |  |
| other | 2 | 0 | 0 | 0 |  |
| **Educational level *N*** |  |  |  |  |  |
| Low | 91 | 80 | 86 | 92 | Χ^2^(3) = 3.40 *p* = .334 |
| High | 35 | 42 | 29 | 31 |  |

N, number of participants; M, mean; SD, standard deviation

^1^ due to low case number ‘other’ were excluded for the statistical test.
