# Supplementary material for: People’s perceptions of, willingness-to-take preventive remedies and their willingness-to-vaccinate during times of heightened health threats
Source: PLoS One. 2022 Feb 2;17(2):e0263351. doi: 10.1371/journal.pone.0263351 (PMC8809555; doi:10.1371/journal.pone.0263351)
Supplement: S2 Table — M, mean; SD, standard deviation; n, sample size per condition; t, t-value; df, degrees of freedom; p, probability of the hypothesis test. (DOCX) [file pone.0263351.s002.docx]

**S2 Table. Independent t-test comparing pandemic health fears for participants in the low and high salience condition.**

|  | Low salience  *n* = 238 | High salience  *n* = 248 | *t* (df), *p* |
| --- | --- | --- | --- |
| Pandemic health fears | 3.98 (1.42) | 3.88 (1.54) | 0.71 (484), .480 |
| Pandemic societal fears | 4.57 (1.30) | 4.54 (1.29) | 0.31 (484), .758 |

M, mean; SD, standard deviation; n, sample size per condition; t, t-value; df, degrees of freedom; p, probability of the hypothesis test.
