# Supplementary figures and images for: People’s perceptions of, willingness-to-take preventive remedies and their willingness-to-vaccinate during times of heightened health threats
Source: PLoS One. 2022 Feb 2;17(2):e0263351. doi: 10.1371/journal.pone.0263351 (PMC8809555; doi:10.1371/journal.pone.0263351)

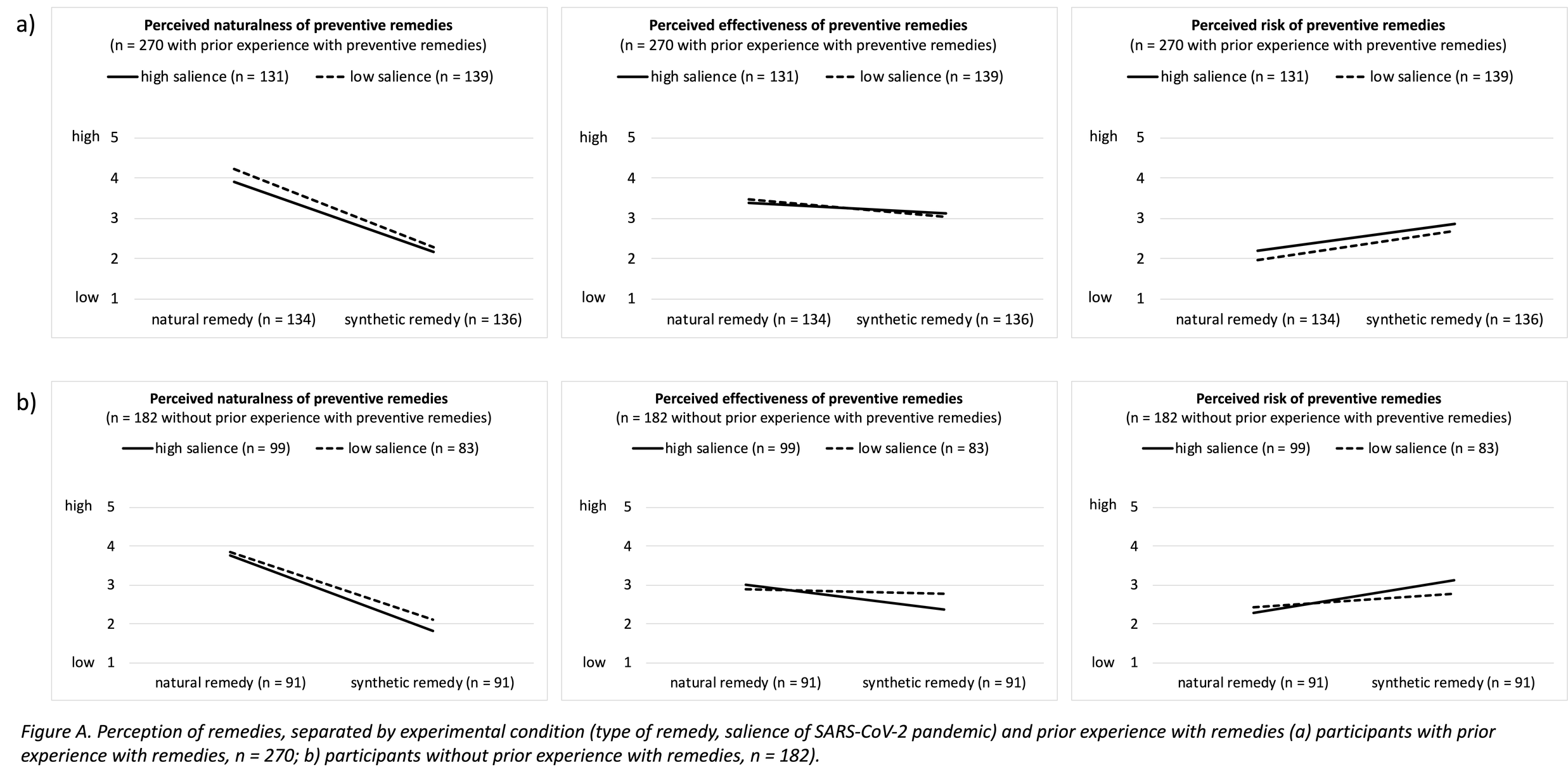

Supplement: S1 Fig — Type of remedy, salience of SARS-CoV-2 pandemic) and prior experience with remedies (a) participants with prior experience with remedies, n = 270; b) participants without prior experience with remedies, n = 182. (TIF) [file pone.0263351.s003.tif]
