# Supplementary material for: People’s perceptions of, willingness-to-take preventive remedies and their willingness-to-vaccinate during times of heightened health threats
Source: PLoS One. 2022 Feb 2;17(2):e0263351. doi: 10.1371/journal.pone.0263351 (PMC8809555; doi:10.1371/journal.pone.0263351)
Supplement: Attachment — Submitted filename: Response to Reviewers.docx [file pone.0263351.s004.docx]

**Response to reviewers**

Reviewer #1: Thank you for the opportunity to provide peer review for the article „People’s perceptions of, willingness-to-take preventive remedies and their willingness-to-vaccinate during times of heightened health threats”.

The well-written article focuses on a sample of 452 individuals in Switzerland and explores their perception of natural or synthetic remedies as well as their willingness to take those remedies when a health threat is made salient. Furthermore, the authors investigated people’s perceptions of vaccines and their willingness to vaccinate against SARS-CoV-2. In the study, the authors tried to identify possible influencing/predicting factors for both the willingness to take remedies as well as for the willingness to vaccinate against SARS-CoV-2. The authors argue that, especially in times of heightened health threats such as the current Covid-19 pandemic, it is important to identify such factors particularly to prevent misinformation and to prevent vaccine hesitancy. The information gained could be used by governments to launch appropriate strategies to increase vaccine acceptability.

Firstly, the study found that even when the actual health threat was made salient the expected curative mind set did not arise and people still preferred natural remedies. Secondly, the authors identified different factors such as expected effectiveness, low risk and perceived naturalness, that were positively influencing on the perception and the willingness to take remedies.
Thirdly, the data shows which factors are associated with people’s willingness to vaccinate.
**Response by authors:** Thanks for taking your valuable time and reviewing our articles. You have raised relevant issues that we are happy to address subsequently and in our revised article. We are thankful that your input has substantially improved our article.

The following points should be considered:

- The authors present “perceived naturalness of a preventive remedy” and later write about “a curative mindset”. This is confusing and likely inappropriate since all approaches in the context of SARS-CoV2 infection are preventive or supportive at best and to the best or our knowledge not curative as such.

**Response by authors:** We agree with the reviewer on this point and had already discussed this point prior to submission as a likely source of confusion. The “curative mindset,” as Scott et al. (2020) called it, states that people will place more weight on higher perceived effectiveness, independent of perceived naturalness. Even preventatives (such as the vaccine) or believe-preventatives (such as synthetic/plant-based remedies that claim to strengthen the immune system) are seen as “curing the pandemic,” which is where the term “curative” in curative mindset originates from. We agree though that the terminology might be misleading in our article and have removed the terminology and clarified where necessary (see changes throughout the paper, whenever we discuss the “curative” mindset).

- Please give a definition for type of remedy natural vs. non-natural, e.g. supplement like vitamin D which may be perceived natural by some and not by others.

**Response by authors:** We added more information on this on page 4 (lines 77-80).

- Please state what the role of the market research company (respondi AG) was in the context of the presented work.

**Response by authors:** the market research company solely supported recruiting by providing us access to their market research panel (we have specified this now in the article on page 11, lines 2237-238)

- Please explain how participants were selected and how representative the population is for the Swiss population.

**Response by authors:** Participants were members of the market research panel mentioned above. Thus, they had previously indicated their willingness to fill out questionnaires for respondi. No other selection criteria were applied. However, quota sampling was used, which is why the sample is representative in terms of sex (49% males in the sample vs. 50% in Swiss population in 2020) and age (mean age of 46 in the sample vs. 43 in the population in 2020). Moreover, the distribution in educational level suggests heterogeneity.

- The participants were randomly assigned to one of four conditions. Please show the characteristic for these conditions separately (e.g. as supplementary appendix)

**Response by authors:** We have added the required information (which shows that randomisation was successful in the measured characteristics). See S1 Table.

- In Table 5 it is not clear which quadrants the authors are referring to and how the results in the table should be interpreted.

**Response by authors:** We have added some additional reader guidance in the text on page 15 lines 332-336.

- Further explanation how the numbers in Table 6 can be interpreted would be appreciated.
**Response by authors:** It is customary to present the bivariate correlations before presenting a linear regression analysis. Therefore, we see Table 6 as a pre-requisite for Table 7. We have added some information on interpreting correlation coefficients on page 12, lines 258-261 and further explanations on page 15, lines 335-337.

For the editor: I could not adequately address the statistical methods.

Minor points:
- Baseline characteristics that were collected (seen in Table 7) should be mentioned in the methods.

**Response by authors:** They were mentioned in the methods but were easy to miss. We have now made this explicit by including the variable names for all variables (see pages 8-11).

- Figure 1: Description of pictures: 1, 2 and 3 instead of a, b and c.

**Response by authors:** Thanks for spotting this mistake. Amended.

- Line 209: Both the scales for pandemic health fears (…)

**Response by authors:** Amended.

- It is unclear if “unnatural/natural” vs. “chemical/not chemical” or “has no effect/has an effect” vs. “ineffective/effective” evaluate something different in Table 1 and 2.

**Response by authors:** Indeed, this is deliberate. These items intend to measure a homogenous latent construct (a theoretical construct that cannot be observed or measured directly). It is standard practice in the social sciences to measure a latent construct (perceived naturalness) with several similar, yet slightly different items (unnatural/natural, chemical/not chemical…) (Bandalos, 2018). The goal is to maximise the internal consistency (correlations of each item within a scale with another item in the same scale, operationalised as Cronbach’s Alpha) of a particular measurement instrument, one of the important quality criteria of a measurement instrument.

- Abbreviation missing for “r” in Table 3, “n” in Table 4 and Table 6 and “n”, “B”, “95% CI”, “Beta” and “p” in Table 7.

**Response by authors:** It is rather unusual to label some of these abbreviations (n, B, p), but as PLOS One is an interdisciplinary journal, we have added this information. Thanks for spotting this.

- It should be stated in the methods which p-value was deemed statistically significant.

**Response by authors:** Included in section 3.3 Data Analysis.

- Write number of excluded persons in parenthesis or total number of remaining persons outside the parenthesis to avoid misunderstanding.

**Response by authors:** This should be clearer now.

- Sentence on lines 467-469 (As our study found the important role of perceived …) is difficult to understand. Consider adjustment.

**Response by authors:** Done.

- The manuscript needs some revision on spelling and punctuation.

**Response by authors:** The manuscript has been spell-checked.

In section 5.1 the authors claim that a curative mindset (which means preference for a more effective drug/remedy) could arise if the perceived risk of a health threat is higher. In prior studies the synthetic drugs/remedies were deemed to be more effective and thus a curative mindset let to a preference of the synthetic drug/remedy. In this study the authors discuss that there is no curative mindset based on the fact that the willingness to take the natural remedy is still preferred over the synthetic one. Considering the fact that in this study the perceived effectiveness of the natural remedies was higher than that of the synthetic remedy (Table 1), the conclusion that there is no curative mindset doesn’t seem logical. The result mean that even if there was a shift towards a curative mindset people in this study

would still choose the natural remedy, as they perceived it as more effective.
**Response by authors:** Thanks for this important point, which we have now addressed in our discussion section (pages 18 and 19).

The number of discussed limitations in the study is limited:
Due to the pandemic during the time of data collection, there was probably no real
low-salience group. Perceived current health threat would have been interesting to try to evaluate if there was a difference between the low salience and high salience group.

**Response by authors:** An important input. We had previously checked whether the two groups (low and high salience) indeed differed in pandemic health and societal fears, which was not the case. We have now added more weight to this information to the paper (Page 19, lines 448-450) and more explicitly in the supplementary information (S2 Table).

No objective test evaluating the knowledge about the vaccines was done. Data based on self evaluation.

**Response by authors:** Measuring objective knowledge about (mRNA) vaccines is a non-trivial task. To the best of our knowledge, no scale has been developed and validated to date that measures objective knowledge of mRNA vaccines. What makes the task of measuring knowledge objectively challenging is to develop knowledge items that measure relevant knowledge (see Siegrist and Árvai (2020) for a discussion about the measurement of objective knowledge). Likely, it is not relevant for people to know what mRNA stands for, whereas it might be relevant to understand what happens in the body when a mRNA vaccine is administered.

Most importantly, as knowledge was not a core construct for our study, we did not include an objective measure. Further, the impact of objective vs. subjective knowledge on willingness-to-vaccinate is not so much about objective vs. self-evaluation. Thinking to know more about vaccines can have an impact on willingness-to-vaccinate above and beyond the actual knowledge a person has (a phenomenon summarised under the term Dunning-Kruger effect (Dunning, 2011)).

For these reasons, we feel that we have already addressed this in our article on page 21, lines 491-510 and that the use of subjective knowledge is not really a limitation.

The study was conducted at an early stage after vaccinations against SARS-CoV-2 was available. This represents the early vaccination hesitancy and maybe not the current situation. A revaluation would be necessary to evaluate the current situation.
Compared to the SAGE-WG Model the authors of this study only include a small number of the numerous mentioned possible determinants of vaccine hesitancy (H. J. Larson et al.; Understanding vaccine hesitancy around vaccines and vaccination from a global perspective, A systematic review. <https://doi.org/10.1016/j.vaccine.2014.01.081>)

**Response by authors:** We fully agree with both points and have added this information on page 22, lines 517-525. Also, we have added the time of data collection in the abstract to make this point salient.

Reviewer #2: Thank you for the opportunity to review the article. This manuscript highlight people's perception regarding natural and synthetic remedies. There are at least two limitations to this manuscript, first the sample size is very small especially during this time of pandemic where literally everyone on the globe is involved, so the denominator actually should be way larger than what assumed to be the targeted population. the second limitation is that there is a time factor in shaping the opinion of people regarding the investigated perspectives, in the beginning of the pandemic people had a lot of misconceptions that were corrected as more information becomes available and reiterated in media.

**Response by authors:** Thanks for reviewing our article. We would like to subsequently address these two points of critique in detail.

Sample size: We disagree with the underlying suggestion that a larger sample size is always better than a smaller sample size. The sample size is not determined by the size of the population, rather a power analysis should be conducted to determine the appropriate sample size. This was done in our case (see page 11, lines 238-239).

There are weighty reasons against conducting this experiment on a global scale. Some of these reasons are related to feasibility (e.g., development of a questionnaire in various languages, costs of data collection). However, we think that the weightier argument is that there exist cultural differences and limits to the validity of questionnaire translations that weaken approaches that cover several countries or continents from a methodological perspective. In this study, we were interested in psychological mechanisms that are suggested to be universal, however, the operationalisation and measurement of these psychological mechanisms is not universal and should be carefully adjusted to the cultural and linguistic context.

Time factor: We agree that we are providing a snapshot of early vaccine perceptions (see also response to reviewer 1) and have now addressed this in the article (see page 21, lines 517-525). This is indeed an important limitation of this study, which should be addressed with future studies (that can build on our foundation). However, we disagree that clearing up misunderstandings is going to universally improve vaccine hesitancy (an oversimplification described in the literature as knowledge deficit model (Hansen, Holm, Frewer, Robinson, & Sandøe, 2003; Simis, Madden, Cacciatore, & Yeo, 2016)). Vaccine hesitancy is a more complex phenomenon, as we have now also included in our discussion of the limitations (see page 22, lines 517-525).

Bandalos, D. L. (2018). *Measurement Theory and Applications for the Social Sciences*. New York: Guilford Publications.

Dunning, D. (2011). The Dunning–Kruger effect: On being ignorant of one's own ignorance. In *Advances in experimental social psychology* (Vol. 44, pp. 247-296): Elsevier.

Hansen, J., Holm, L., Frewer, L., Robinson, P., & Sandøe, P. (2003). Beyond the knowledge deficit: recent research into lay and expert attitudes to food risks. *Appetite, 41*(2), 111-121. doi:<https://doi.org/10.1016/S0195-6663(03)00079-5>

Siegrist, M., & Árvai, J. (2020). Risk perception: Reflections on 40 years of research. *Risk Analysis, 40*(S1), 2191-2206. doi:10.1111/risa.13599

Simis, M. J., Madden, H., Cacciatore, M. A., & Yeo, S. K. (2016). The lure of rationality: Why does the deficit model persist in science communication? *Public Understanding of Science, 25*(4), 400-414. doi:10.1177/0963662516629749

Zingg, A., & Siegrist, M. (2012). Measuring people's knowledge about vaccination: developing a one-dimensional scale. *Vaccine, 30*(25), 3771-3777. doi:10.1016/j.vaccine.2012.03.014
